# Contemplative self healing in women breast cancer survivors: a pilot study in underserved minority women shows improvement in quality of life and reduced stress

**DOI:** 10.1186/1472-6882-14-349

**Published:** 2014-09-23

**Authors:** Mary E Charlson, Joseph Loizzo, Alyson Moadel, Miles Neale, Chayim Newman, Erin Olivo, Emily Wolf, Janey C Peterson

**Affiliations:** Division of Clinical Epidemiology and Evaluative Sciences Research, Center for Integrative Medicine, Weill Cornell Medical College, 338 East 66th Street, New York, NY 10065 USA; Nalanda Institute for Contemplative Science, Weill Cornell Center for Integrative Medicine, Columbia University Center for Buddhist Studies, 300 Central Park West, 1D, New York, NY 10024 USA; Albert Einstein College of Medicine, Jack and Pearl Resnick Campus, 1300 Morris Park Avenue Belfer Building, Room 1006, Bronx, NY 10461 USA; Ferkauf Graduate School of Psychology, Yeshiva University, 1165 Morris Park Ave, Bronx, NY 10461 USA

## Abstract

**Background:**

Among underserved, largely minority women who were breast cancer survivors, this pilot project was designed to evaluate the quality of life outcomes of a 20 week Contemplative Self-Healing Program.

**Methods:**

Women previously treated for stage I-III breast cancer were assessed before and after the 20 week program with the FACT-G, FACT-B, FACIT-Spirituality, ECOG, and the Impact of Events Scale. They participated in a 20-week intervention involving guided meditation and cognitive-affective-behavioral learning.

**Results:**

With an average age of 63, 62% of the participants were African-American or Latino. With an average of 5.4 years since the diagnosis of breast cancer, 72% had an ECOG performance status of 1. 57% were currently working.

Their baseline FACT-G was 80.5 ± 15.1, and their baseline Impact of Events Scale was 26.3 ± 18.9. The within-patient improvement on the FACT-G was 4.6 ± 10.9 (p = .01); in parallel the FACT-B improved by 2.8 ± 12.8 points (p = .03). The Impact of Events Scale improved by 6.6 ± 15.5 points (p = .01). There was significant within-patient improvement on both the avoidance scale (3.8 ± 9.2) and on the intrusion scale (2.9 ± 7.9). Patients who attended more sessions and conducted more home practice had greater improvements in quality of life.

**Conclusion:**

Persons receiving a 20-session contemplative self healing intervention showed improved quality of life, with a clinically and statistically significant increase in the FACT-G. In addition, this population showed a significant reduction in post-traumatic stress symptoms assessed by the Impact of Events Scale.

**Trial registration:**

Clinical Trials Gov NCT00278837.

## Background

Even after five years, when most other cancers are viewed as cured, women with breast cancer face an ongoing threat of recurrence, at about 5% per year [1, 2]. While it is often assumed that the stress experienced by women with breast cancer abates after initial treatment, paradoxically, stress-related symptoms may increase when treatment is finished and they leave the ‘safety net’ provided by their oncologist and their staff [3]. Patients may experience feelings of uncertainty as they try to reintegrate themselves back into their pre-diagnosis lives and roles [3]. If patients cannot address and resolve such feelings, they may experience persistent stress symptoms [3, 4]. Thirty to forty percent of breast cancer survivors experience distress and stress-related symptoms that do not abate with time [1]. In fact, twenty years after treatment, women who are disease-free may experience ongoing distress [1].

In a large pilot study of 50 predominantly white, college educated, and with higher socioeconomic status breast cancer survivors, we previously demonstrated that a contemplative self-healing intervention resulted in a clinically significant improvement in within-patient differences in quality of life, using the FACT-G, a cancer-specific scale [5]. This contemplative intervention was based on two thousand year old practices of healing visualization and lifelong health education preserved in the Tibetan Buddhist traditions of integrative medicine and universal public health education [6]. The visualization-based health educational practice incorporates an imagery-based mindfulness practice for meditation-based stress reduction with an imagery-based contemplation practice for cognitive-affective-behavioral learning [6]. Since underserved and minority breast cancer survivors have more stress and lower quality of life [3, 7–13], this study was designed to assess the impact of the contemplative intervention on minority cancer survivors.

Among underserved, predominantly minority women who had completed treatment for stage I-III breast cancer, the objective of this pilot study was to evaluate the impact of our Contemplative Self-Healing Program on quality of life (FACT-G, FACT-B) and post-traumatic stress symptoms.

## Methods

### Screening and eligibility

The Montefiore-Einstein Center for Cancer Care (MECCC) provides cancer care to residents of Bronx County, NY and surrounding areas. The poorest urban county in the U.S., the Bronx consists of nearly 1.4 million residents, most of whom are members of ethnic minorities. The primary racial/ethnic groups represented in the Bronx are Hispanics (51%), Blacks (34%), and non-Hispanic Whites (22%), with 30% living at or below the poverty level.

Psychosocial Oncology program staff selected potentially eligible participants, who were then contacted over the phone and screened for eligibility by study personnel. Women with stage I-III breast cancer who had completed initial treatment were eligible for inclusion in this program. Patients who were male, were known to have stage IV (metastatic) breast cancer, or were unable to return to the MECCC Center for classes were excluded. Women who were not fluent in English, those enrolled in other studies involving psychosocial interventions for breast cancer survivors, or those with cognitive impairment were excluded. The study was approved by the Weill Cornell Medical College Institutional Review Board and written informed consent was obtained.

### Baseline evaluation

#### Demographic and clinical status

Baseline evaluation included data about demographics, acculturation [14], and living situation. Clinical information— including the cancer stage, axillary node status, estrogen/progesterone receptor status, HER-2, and specific course of chemotherapy, radiation, and surgery—was obtained from clinical records. Comorbidity was assessed with the Charlson Comorbidity Index [15]. Cognitive function was measured through the EORTC cognitive function questionnaire [16]. The Eastern Cooperative Oncology Group Performance Status Rating was assessed. (ECOG PSR) [17]. The patient’s smoking history, physical activity, nutritional, and other lifestyle practices were also documented.

#### Psychosocial impact of disease

The Impact of Events Scale (IES) was used to assess post traumatic stress symptoms on two subscales: intrusion and avoidance with higher scores indicating a greater number of post traumatic stress symptoms [18]. The IES provides a dimensional measure of severity, rather than the DSM categorical assessment. However, IES Items correspond directly to 14 of the 17 DSM-IV symptoms of PTSD [19].

#### Quality of life outcomes

Quality of life was measured by the general Functional Assessment of Cancer Therapy Scale (FACT-G, Version 4) [20]. The FACT-G measures quality of life, evaluating 28 items across four domains: physical well being (7 items); social/family well-being (7 items); emotional well-being (5 items); and functional well-being (7 items). A higher score indicates higher Quality of Life. In addition, the Breast Cancer Subscale, which has disease-specific questions including treatment sequelae, was administered [20, 21]. The BCS and FACT-G are added to form the FACT-B. The FACIT Spirituality scale was also given [22, 23], with a range of 0–48 [22]. The 9-item Ryff scale was also administered; this scale assesses psychological well-being across multiple distinct dimensions: autonomy, environmental mastery, personal growth, positive relations with others, purpose in life, and self-acceptance [24–26].

#### Program completion

All quality of life and psychosocial scales were repeated at program completion. Patients were also asked about any interval medical events. Finally, patients were asked open-ended questions about their experience with the program, what they would tell others about the program, and what the program effects for them.

### Contemplative self-healing intervention

The intervention program is a 20 week meditation-based stress reduction program organized in two parts [6]. The first part consists of eight weekly 90-minute group sessions focus on teaching meditation skills. During these sessions, participants were guided through exercises emphasizing awareness of breath, healing imagery, and deep breathing to help them better control their responses and practice self-change. The second part of the course was a 12-session cognitive-affective-behavioral learning program. Participants were exposed to new ideas and tools to better understand and cope with their reactions to the challenges of living with illness. The weekly sessions emphasized mind/body skills to help participants unlearn disease-prone habits and develop a healthier outlook, attitude, and lifestyle.

Daily practice was supported by homework consisting of visualization guided by audio tapes and structured assignments for reading and reflection. There were four separate groups held in 2008-2009--two offered in the morning and two in the evening. The sessions were led by four different interventionists who were trained by its developer (J.L.). The four interventionists were all psychologists – two with extensive experience and two still in training. Two of the four leaders had no experience with contemplative group interventions and none had previously led this intervention. There was ongoing supervision throughout the study.

### Data analysis

All data was entered into a web-based, secure, HIPPA compliant data management system, Clinvestigator©. Data analysis was conducted using SAS 9.2. Bivariate differences were assessed by Chi-square for differences in proportions and the Student-paired *t*-test was used to evaluate within-patient differences in scores. PROC GLM in SAS 9.2 was employed for regression analysis.

Qualitative data was analyzed using Grounded theory methods [27, 28]. Similar concepts were grouped and categories with similar properties and dimensions defined until data saturation was attained [27, 29]. We then identified common themes. Data was analyzed an experienced qualitative research investigator (JCP) and corroborated by a second reader (MEC) to establish trustworthiness [30].

**Figure 1:**
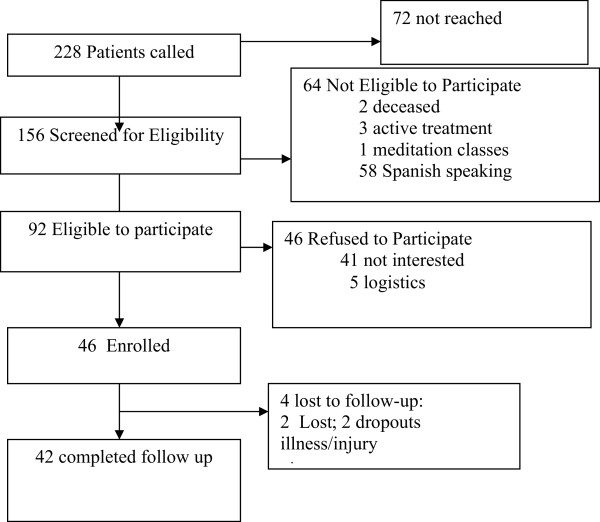
**CONSORT flowchart.**

The Consort Diagram (Figure 1) shows the assembly of the population studies.

## Results

A total of 46 women consented to participate and provided informed written consent. Of those 46, four of the patients (10%) were lost to follow-up. They were more likely to be primary Spanish speakers (p = .0005). There were no other baseline differences in FACT-G, Ryff, or IES scores between those who were lost and those who were not.

### Sociodemographic and clinical characteristics of participants

Half were Black (African American, Caribbean, or African), 7% were Latino, 29% were Caucasian, and 5% were Asian (Table 1). Their average age was 63 years, with a range of 42 to 81. Only 2% were primary Spanish speakers. 67% of participants had completed college. With regard to religion, 64% were Protestant (from a variety of denominations), while 31% were Catholic. The average time since the patient’s initial breast cancer diagnosis was 5.7 years. 4% had experienced a recurrence before study enrollment. Most patients had excellent ECOG status. According to the EORTC-cognitive function scale, 12% had a lot of difficulty concentrating or remembering things.

**Table 1:** **Baseline demographic and clinical characteristics of the participants in the Contemplative Self Healing Program**

	Completed (N = 42)
**Age** (mean years ± SD)	63 ± 11
**Race**	
Caucasian	29%
African American	55%
Latino	7%
Asian	5%
**Primary language Spanish**	2%
**Marital status**	
Married	41%
Separated/divorced/widowed	40%
Single	17%
**Lives alone**	29%
**Finished college**	67%
**Working**	57%
**Retired**	40%
**Religion**	
Protestant	64%
Catholic	31%
Daily prayer	79%
**Time since breast cancer diagnosis** (years ± SD)	5.7 ± 5.8
**Positive nodes**	38%
**Breast cancer treatment**	
Surgery	98%
Chemotherapy	67%
Radiation	55%
Hormonal therapy	41%
Current hormonal therapy	41%
**Previous breast cancer recurrence**	4%
**ECOG Performance Status**	
Fully active	72%
Limited strenuous activity	21%
Ambulatory but unable to work	7%
**EORTC cognitive**	
No difficulty (2–3)	47%
Some difficulty (4–5)	41%
A lot of difficulty (6–8)	12%
**Health behaviors**	
Current smoker	10%
Ever smoked	38%
**Needs to cut back alcohol**	9%
**Sleep**	
>6 hours a night	63%
4-5 hours a night	12%
<4 hours a night	15%
**Ever meditated before**	31%
Few times a week to daily	7%
Less than once a week	17%
**Obese**	26%
**Comorbidity**	
1-2	67%
3-4	26%
≥5	7%
Hypertension	36%
Diabetes	26%
CVA	2%
Asthma	20%
Other cancer	4%
Anti-depressants	14%
**Stressors**	
Stress with health	39%
Stress with relationships	29%
Stress with job	29%
Stress with money	41%
Other stresses	32%
**FACIT Spirituality**	38.9 ± 7.0
**Ryff Personal Well Being Scale**	
Autonomy	44.6 ± 6.5
Environmental mastery	42.9 ± 6.2
Personal growth	42.9 ± 7.2
Relations	43.2 ± 7.5
Purpose in life	40.3 ± 6.6
Self acceptance	41.2 ± 6.8

The baseline FACT-G score was 80.8 (the principal outcome) almost identical to the average FACT-G for the general population and for cancer survivors [31]. The scores for FACT-G subscales -- emotional, physical, social, and functional well being -- were also almost identical to the general population. The Breast Cancer Subscale score (BCS) was 24.3, similar to the BCS reported for other breast cancer patients [21]. The baseline FACIT spirituality was 38.9, similar to scores previously reported [21]. Before starting the program, 17% of participants had meditated more than a few times a week and 79% prayed daily. Of note, the baseline FACT-G score was significantly was not significantly associated with prior recurrence (p = .26), although it was lower in patients with higher comorbidity (p < .02) and higher in patients with a higher FACIT spirituality score (p < .001). The scores on the Ryff Personal Well Being were similar to those previously reported for breast cancer survivors and healthy controls [32]. Therefore, with regard to quality of life and personal well being at baseline, the participants had comparable scores both to women of a similar age and to other women who were cancer survivors. At baseline, 37% reported one or two stresses, and 34%, three or more, attributed equally to health and money followed by relationship and job stresses. On the Impact of Events Scale, our patients had 15.0 for the avoidance scale and 11.0 for the intrusion scale, similar to other reports for early stage breast cancer patient [33, 34].

### Principal outcome

The principal outcome was the FACT-G which focuses on overall quality of life. Among all participants, the FACT-G improved by 3.7 points after completion of the program (p = .04), which is a clinically important improvement in FACT-G [35, 36]. Importantly, three women developed a **new** recurrence of breast cancer *during* the 20-week program; not surprisingly, they had a worsening of 7.7 on the FACT-G. The remainder of the analysis will focus on patients without an interval recurrence.

### Outcomes among patients without interval recurrence

#### Quality of life

The 39 women who did not have a recurrence during the 20 week program had a clinically and statistically significant improvement of 4.6 in the FACT-G (p = .04). Table 2 displays within-patient differences for the subscales for the FACT-G. There was a significant improvement in social well being (p = .03), but not in the other subscales. The BCS scores, which measure aspects of quality of life specifically related to breast cancer (e.g. arm edema), did not significantly change (p = .83). The FACIT-Spirituality scale was also not changed (p = 0.17).

**Table 2:** **Outcomes in breast cancer patients without interval recurrence (n = 39)**

Outcomes	Before program	After program	Within patient change	Paired ***t***-test
**FACT-G**				
Total	80.5 ± 15.1	85.1 ± 14.0	+4.6 ± 10.9	.01
Emotional	18.5 ± 5.0	19.3 ± 4.3	0.8 ± 4.1	.21
Physical	22.8 ± 4.2	23.5 ± 4.2	0.7 ± 3.5	.25
Social	20.1 ± 6.3	+21.7 ± 4.9	+1.6 ± 4.6	.04
Functional	19.1 ± 6.6	20.5 ± 5.5	+1.4 ± 4.5	.06
**BCS**	24.3 ± 6.6	24.5 ± 6.5	+0.2 ± 5.2	.83
**FACT-B**	104.9 ± 19.9	109.6 ± 18.6	+4.8 ± 12.8	.03
**FACIT spirituality**	37.2 ± 8.2	38.9 ± 7.1	+1.4 ± 1.0	.17
**Impact of events**				
**EvEvents**				
IES Total	26.3 ± 18.9	19.8 ± 15.5	−6.6 ± 15.5	.01
IES avoidance	15.5 ± 10.6	11.4 ± 8.1	−3.8 ± 9.2	.01
IES intrusive	11.5 ± 9.3	8.4 ± 8.3	−2.9 ± 7/9	.03

#### Post-traumatic stress symptoms

The total score on the Impact of Events Scale (IES) improved by 6.6 (p = .01), with an improvement of 3.8 for avoidance (p = .01 and 2.9 for intrusion subscale (p = .03).

#### Other health measures

Between baseline and follow-up, there were minimal changes in other health behaviors. There were no reported changes in number of hours of sleep, weight, or smoking. For the most part, physical activity remained the same. There were also no significant changes in any of the Ryff Well Being Subscales. ECOG performance status was the same in 71% of patients, improved by a rank of one in 16% of patients, and worse in 13% of patients. There was also a significant improvement in the cognitive domain of the EORTC (p < .0001); 43% of the women (17/39) reported an improvement in thinking or concentration by at least a rank of 1, while 28% reported an improvement by a rank of 2 or more.

### Program adherence

Overall, 7 participants (18%) attended 1–4 classes; 6 (15%) attended 5–9 classes, and 22 (56%) attended 10 or more classes. Those who attended 5 or fewer sessions reported a variety of reasons, including work, the distance to the classes, difficulty hearing, death in the family, and injury. The magnitude of the change in FACT-G was related to the number of class sessions attended and the frequency of meditation at home (Table 3). Those who attended ten or more classes and who practice meditation at home on most or every day had the greatest improvement in FACT-G. Of those who meditated at home, about half used a tape or CD and half did not. There were similar findings for IES; those who attended 10 or more classes and meditated most or all days showed a decrease in total IES score of 7.7, while those who attended 10 or more classes and who meditated less frequently showed a decrease of 3.6. Multivariate analysis revealed that the principal predictors of FACT-G at program completion were baseline FACT-G (p=.001), EORTC (p = .001), as week as the number of classes attended (p = .02). Meditation practice was not significant (p = .09). Baseline ECOG performance status was a significant predictors of IES scores at completion (p = .005); but baseline IES, number of classess attended and meditation practice were not.

**Table 3:** **Changes in FACT-G and IES according to program participation: Class attendance and meditation at home**

	Within-patient change in FACT-G	Within patient change in IES
Class attendance		
1-9 classes		
Meditation never, rarely or some days	+3.3 ± 10.3	−7.0 ± 21.4
Meditation most days or every day	+5.5 ± 4.9	−5.5 ± 21.9
10-20 classes		
Meditation never,rarely or some days	+5.1 ± 11.4	−3.6 ± 13.2
Meditation most days or every day	+6.5 ± 13.5	−7.7 ± 15.2

### Qualitative results

Three themes emerged from the qualitative analysis of the participant’s experience (Table 4). First, the classes provided skills (i.e., meditation, breathing) as well as a positive supportive environment that the women found helpful and enhanced their ability to cope with the uncertainty of living as a cancer survivor. Second, participants reported a new and positive outlook on life, with feelings of empowerment, competence, personal strength, and an overall enhanced sense of control. Third women reported decreased stress and anxiety and a greater sense of calm, serenity and balance. This was associated with increased acceptance/understanding of self and others and a decrease in worrying about dying.

**Table 4:** **Comments of participants**

**What was your experience?**	I really found it much better than I truly anticipated.... When people brought things up it was relatable. It brought things to light and clicked like a puzzle put in.
	I want to know why I didn’t know about something like this before. If I had this earlier in my diagnosis and after my surgery it would have helped a lot. I would have taken a different turn.
	I was able to talk and get all these things out of me to make myself calmer. It helps me to relieve myself and relax.
	I found myself honored and blessed to be in the company of my cohort and facilitators. We come from disparate backgrounds, yet we experience a one-ness that was nothing less than divine.
**What was its effect?**	I used to be so angry but since this I’ve been so mellow. I have to worry about my sick husband and not myself and I one time felt like just packing up and running away from everyone. Until I listened to these CDs and reading and just meditated and listened.
	Positive, empowering, emotionally freeing. It took a couple of rocks from my bag.
	The change in the way we feel about ourselves. It helps you w/every aspect of your life. Sometimes you see some of those ladies with breast cancer and they feel so sorry for themselves. They don’t realize the power they have to heal themselves.
	I knew nothing before and I had a lot of stress and it helped me be more positive. It taught me to take my time to think before acting. I think about giving people a chance.
	I’m less anxious and more relaxed and it helped me realize when I’m anxious and then I use what I’ve learned.
	I don’t worry about dying as I did before when it was a lingering thing. I have a new out look now with the meditation. I do need that meditation. It made me a new person in dealing with my cancer. It helped me to get off and slow the spinning wheel.
	Made me stronger and less critical of myself more accepting of myself.
	In a good way. I feel more competent that I could do whatever I want and that nothing is impossible.
	It was great bcs I was able to go to the past and it wasn’t as bad as I thought it was going to be. I brought it to the future and it wasn’t as scary. It helped calm me down so I can get my life in order and not worry about dying.
**What would you tell others?**	That it would be good and it helps to deal with the stress--how to deal.
	I would tell everyone that they would learn about taking away your stress and anger. The teacher was very patient.
	It helps you to relax and to understand more about yourself and your illness.
	That they should go; they should make the time. There are many benefits.
	Very helpful with everyday life even if you’re not sick it helps you with everyday living.
	That it was quite helpful in concentration, learning to meditate, bring your body into one.
	It’s very good. I recommend it to cancer patients and any other program. It helped me immensely.
	Maybe doctors could learn from this. They could learn something for their patients. It’s helpful and it’s a worthwhile program.
	I would tell them how helpful it is with not just your physical situation but also the general situation.

## Discussion

The overall quality of life of women with breast cancer is directly related to whether they have ongoing stress-related symptoms [1]. Such stress–related symptoms have a significant adverse impact on quality of life and function, especially emotional wellbeing [1]. Stress-related symptoms include intense fear, avoidance, intrusive thoughts, impaired sleep, difficulty concentrating, increased irritability and apprehensiveness—symptoms primarily associated with fear of recurrence [4, 37–39].

We believe that the stress and distress experienced by breast cancer patients is a relatively normal reaction to a life-threatening event. Breast cancer forces women to face life’s impermanence, as well as its meaning and purpose [40]. This may explain why such distress is often not relieved by the passage of time [41–45]. Moreover, the distress is not less among patients who have excellent outcomes [4, 46, 47], because these women still have an increased sense of vulnerability and loss of control [45, 48]. Quality of life and stress-related conditions do improve for those patients who have an enhanced sense of purpose after experiencing breast cancer [49, 50].

### Findings in context

With a few exceptions, most conventional interventions--including professional counseling [51], peer support [52], and telephone support groups [53, 54]—have not improved quality of life or reduced stress–related symptoms in breast cancer patients. Cognitive behavioral therapy has had modest benefits [34, 55, 56]. The only intervention that has markedly improved quality of life for breast cancer survivors is aerobic exercise; the exercise group improved by 5.0 more than the control as measured by within-patient change in the FACT-G (+5.7 vs. +0.7) [54]. This intensity of aerobic exercise, three times a week for 15 weeks [54], is also as effective as anti-depressant medications in treating major depression [57]. Lesser levels of supervised exercise or home-based physical activity were not effective. In our previous study involving primarily Caucasian patients [6], the improvement in overall FACT-G was slightly greater with an improvement of 6.4 vs. 4.6 in the current study. This is remarkable because women in this study reported a lower level of adherence regarding meditation. Since individual levels of improvement in this study were correlated with class attendance and practice, increased adherence might enhance the intervention effect.

There is limited evidence that *any* intervention in breast cancer patients has been efficacious in decreasing post-traumatic stress related symptoms. In contrast, the qualitative and quantitative results of our contemplative self-healing intervention resulted in improved quality of life and stress-reduction ratings—a significant potential benefit to be analyzed further in future randomized trials.

A secondary outcome in this study is an assessment of post-traumatic stress symptoms using the Impact of Events Scale (IES) [18]. Post-traumatic stress symptoms have adversely impacted quality of life in breast cancer survivors [47, 58, 59]. Several observational studies of breast cancer patients using the IES suggest that stress-related symptoms improved over time, but did not resolve completely (Table 5). These studies help to provide a context within which our results can be evaluated, since we did not have a control group. One study found over six months after initial surgery, there was a 2.5 point between patient improvement in total IES, less than half of the improvement in this study [60]. The largest study assessed 3318 Danish women three months after surgery for breast cancer [58] and found that patients had a 3.9 point improvement after 12 months, also less than this study. In both, our patients showed greater improvement in avoidance. In addition, one RCT evaluated a cognitive behavioral stress management intervention that did not result in differences in IES vs. a control group [34]; however, after 9 months the IES total score improved by 4.4 points, again less than this study.

**Table 5:** **IES total and subscale scores in longitudinal studies of breast cancer patients**

	Between patient changes [60]			Between patient changes [58]			Changes [34]		
Type of study	Observational			Observational			RCT		
Initial assessment	Baseline new surgery	Six months after baseline		Baseline: 3 months after surgery	12 months after baseline		Baseline: 2 months after surgery	9 months after baseline	
N	117			3318		−3.9			
IES Total	24.2	21.7	−2.5	20.1	16.2	−1.6			
Avoidance	12.9	12.1	0.8	10.0	8.4	−2.3	15.6	14.0	1.6
Intrusion	11.4	9.6	1.8	10.1	7.8		16.0	13.2	2.8

Our qualitative results demonstrated that participants benefited from participating in a positive, supportive group format that enhanced their ability to cope with cancer. In a qualitative study of 66 African American women with breast cancer, support networks and spirituality were both important coping mechanisms reported by participants [61]. The women in our study also reported feelings of empowerment and personal strength. Finally, our participants told us that the group provided them with a sense of balance and less preoccupation with fear of dying. Such existential fears are common among female breast cancer survivors [62]. Our intervention deals directly with fear of death, and thus we hypothesize that participants were able to work through fears that they may have been unable to discuss with their family and oncologists.

The principal limitation is that we conducted this pilot study without a control group. As a result, we cannot definitively conclude that the results of the intervention differed from results that would be produced in a control. However the findings of this pilot study suggest important potential benefits for contemplative self-healing methods. Previously, only intensive aerobic exercise had a clinically significant impact on improving quality of life as assessed by the FACT-G, our principal outcome, but these exercise interventions are very difficult for most patients to adopt and sustain. The FACT-G was selected as the principal measure, because it more accurately reflects overall quality of life, than the BCS.

## Conclusion

In conclusion, among predominantly Black and Latino women, the 20-week Contemplative Self healing intervention had a clinically important improvement in FACT-G. Significantly, it also had a clinically important impact on post-traumatic stress symptoms, especially avoidance, as measured by the Impact of Events Scale.
